# Comparison of Nutritional Quality and Functional Active Substances in Different Parts of Eight Lotus Seed Cultivars

**DOI:** 10.3390/foods13152335

**Published:** 2024-07-25

**Authors:** Xueting Liu, Wanyu Dong, Yang Yi, Limei Wang, Wenfu Hou, Youwei Ai, Hongxun Wang, Ting Min

**Affiliations:** 1 College of Food Science & Engineering, Wuhan Polytechnic University, Wuhan 430023, China; lxt666662022@163.com (X.L.); dongwanyu0714@163.com (W.D.); yiy86@whpu.edu.cn (Y.Y.); hwf407@163.com (W.H.); aywlinyun@126.com (Y.A.); 2 Hubei Key Laboratory for Processing and Transformation of Agricultural Products, Wuhan Polytechnic University, Wuhan 430023, China; 3 School Biology & Pharmaceutical Engineering, Wuhan Polytechnic University, Wuhan 430023, China; wanglimeiyx@163.com (L.W.); wanghongxunhust@163.com (H.W.)

**Keywords:** lotus seeds, cultivar, sensory quality, functional activity, alkaloids

## Abstract

In this study, “Honghu White Lotus”, “Red Lotus (HH)”, “Hunan Cunshan Lotus (CS)”, “Wuyi Xuanlian”, “Space Lotus 36”, “Fujian Jianning White Lotus (JB)”, “Jiangsu Yangzhou Lotus (JY)”, and “Suzhou Dongshan Lotus” were selected as experimental subjects. The lotus seed flesh and lotus plumule of each cultivar were selected for nutritional quality and functional active substance analyses. Comparing different cultivars of lotus seeds, the protein and crude fat contents of JY flesh were the highest at 65.59 mg/g and 13%, respectively. The VC content of JB flesh and lotus plumule is the highest at 41.56 mg/g and 204.29 mg/g, respectively. JB flesh has the lowest soluble sugar content, at 17.87 mg/g, while HB’s lotus plumule and flesh have the highest content, at 33.67 mg/g and 29.62 mg/g, respectively. There was no significant difference in the crude fat content of the flesh and lotus plumule among the eight cultivars. TK flesh and lotus plumule have the highest amylose content, at 23.67 mg/g and 76.81 mg/g, respectively. Among them, the total starch content of JB (476.17 mg/g) was relatively high, whereas its amylose content was only 26.09 mg/g. Lower amylose content makes it less prone to aging. The total phenolic and flavonoid contents of the JY lotus plumule were the highest, at 18.64 and 21.04 mg/g, respectively. The alkaloid content of CS, HH, and JY was relatively high at 20.01, 19.29, and 18.68 mg/g, respectively. These can provide a consultation for the estimation and processing of the nutritional quality of different lotus seeds.

## 1. Introduction

Lotus seeds (*Nelumbinis Semen*), also known as lotus rice, belong to the water lily family and are mainly distributed in Hubei, Hunan, Fujian, Jiangxi, and other regions of China [1]. In 2023, the annual production of lotus seeds in China reached 2 × 10^8^ kg. Lotus seeds are a medicinal and food resource that is deeply loved by consumers. They not only contain an abundance of nutrients such as proteins, vitamins, and carbohydrates [2] but also contain functional active substances such as flavonoids [3] and phenolics [4]. In addition, lotus seeds contain a large number of alkaloids, such as liensinine, isoliensinine, and neferine, which have the effects of lowering blood pressure, antioxidation, anti-arrhythmias, and cancer prevention [5,6,7].

Recently, market demand for lotus seeds has increased. As a characteristic economic resource for China’s exports [8], it is necessary to preserve or process them to achieve high-quality exports. However, owing to differences in cultivar, origin, and environmental factors, lotus seeds have different appearances, nutritional qualities, and functional active substance contents, which affect their preservation and processing [9]. Appearance and taste are the main indicators of the export trade. Yang et al. demonstrated differences in the content of flavor substances among different cultivars of lotus seeds, with characteristic flavor substances such as amylose and flavonoids, whereas starch content was highly correlated with soluble sugar and water contents [10]. Guo et al. found that there are differences in amino acid, protein, and starch contents among different cultivars of lotus seeds, and the starch particle sizes of lotus seeds with different maturity levels of the same cultivar were also different [11]. In addition, different levels of polyphenols, alkaloids, and enzymes, such as PPO in lotus seeds, can affect the quality of lotus seeds after deep processing. Consequently, the nutritional quality and functional active substances of the lotus seeds are influenced by the cultivar. Differences in nutritional and functional active substances were found among different varieties and parts of the plants. Zhu et al. studied the differences in phenolic compounds and antioxidant activity in different parts of lotus seeds and rhizomes. The total phenolic content (10.77 ± 0.66 mg gallic acid equivalents (GAE)/g_fresh weight(f.w.)_), flavonoid content (1.61 ± 0.03 mg quercetin equivalents (QE)/g_f.w._), and DPPH free radical scavenging rate (9.66 ± 0.10 mg ascorbic acid equivalents (AAE)/g_f.w._) were the highest in lotus plumule [12].

The main chemical components of lotus seed flesh and plumule may differ. Zhang et al. indicate that the flesh of lotus seeds is rich in nutrients such as starch and protein, while the plumule of lotus contains alkaloids and bioactive substances [13]. At present, most studies have focused on the morphological structure, physicochemical properties, gelatinization properties [14], and changes in nutrients during the storage of lotus seed flesh starch [15], while there is relatively little research on the differences in nutritional quality and functional active substances between different cultivars of lotus seed flesh and plumule. Consequently, it is important to determine the differences in nutritional quality and functional active substances between different cultivars of lotus seeds and their different parts.

This study measured the content of nutrients and functional active substances in the flesh and plumule of eight cultivars of lotus seeds, including Hubei Honghu White Lotus (HB), Hong Lotus (HH), Hunan Cunshan Lotus (CS), Wuyi Xuanlian (WX), Space Lotus 36 (TK), Fujian Jianning White Lotus (JB), Jiangsu Yangzhou Lotus (JY), and Dongshan Lotus (SD), to identify the main differences among different cultivars and parts, providing a reference for the further evaluation of lotus seed nutritional quality and processing.

## 2. Materials and Methods

### 2.1. Plant Material

Eight lotus seed varieties (HB, HH, CS, WX, TK, JB, JY, and SD) were obtained from Taobao’s self-operated store. The entire fresh lotus seeds (including flesh and plumule) were picked and dried in August 2023, and the flesh and plumule were separated and ground. The samples were sieved through an 80-mesh sieve, and were stored in a cool sealed glass container until further analysis was conducted.

### 2.2. Measurement of Total Phenol and Flavonoid Content

The total phenol content was slightly modified according to the method of Singleton et al. [16]. Lotus seed flesh (0.5 g) and lotus plumule (0.5 g) powder separately were added to 10 mL of 80% ethanol (*v*/*v*), shaken for 2 min, centrifuged at 10,000 r/min for 20 min at low temperature, and the supernatant was collected. Typically, 0.1 ml of supernatant was mixed well with 0.4 mL of the phenol reagent. After standing for 8 min, a 7.5% (*w*/*v*) Na_2_CO_3_ solution (1.5 mL) was added, and shaken well. The absorbance at 760 nm after 1 h of light avoidance reaction was measured. Distilled water and the reaction reagents were mixed as blank controls, with three parallel sets for each group. Gallic acid was used as the standard curve, and the total phenolic content of the flesh and lotus plumule was expressed as gallic acid equivalents.

The total flavonoid content was slightly modified according to Liu’s method [17]. Lotus seed flesh (0.5 g) and lotus plumule (0.5 g) powder separately were added to 10 mL of 80% ethanol (*v*/*v*), shaken for 2 min, sonicated for 30 min, centrifuged at 10,000 r/min for 20 min at low temperature, and the supernatant was collected. Typically, to 1 mL of each supernatant, 1 mL of NaNO_2_ was added and left for 6 min. Subsequently, 1 mL of Al(NO_3_)_3_ and 10 mL of NaOH were added, and the volume was made up to 25 mL with 80% ethanol. The mixture was mixed and the absorbance was measured at 500 nm after 15 min of reaction. Rutin was used as the standard curve, and rutin equivalents were used to represent the flavonoid content.

### 2.3. Measurement of Soluble Sugar Content

The experimental method of Yang et al. was followed with slight modifications [10]. Lotus seed flesh (0.5 g) and lotus plumule (0.5 g) powder were accurately weighed, 5 mL of distilled water separately was added, and boiled for 30 min. Then, the mixture was centrifuged at 5000 r/min for 15 min, and the precipitate and supernatant were collected. Distilled water (5 mL) was added, and boiled again. The two supernatants were combined and made up to 100 mL volume with distilled water. Diluted 0.5 mL of extraction solution fourfold and 1 mL of 90 g/L phenol solution were mixed well into the solution. Subsequently, 5 mL of concentrated sulfuric acid was added within 5–20 s, and mixed well. Absorbance values were measured at 500 nm after 30 min of standing, and repeated three times for each sample. Sucrose solution was used as the standard curve. The results are expressed as mass fractions (%) using the following formula:

(1) $$\mathrm{Soluble}\;\mathrm{sugar}\;\mathrm{content}\;(\%)=\frac{10^{-6}\;\times \;\rho \;\times \;Ν\;\times \;V}{m}$$

where *ρ* is the mass concentration of the soluble sugars calculated based on the standard curve (μg/mL), V is the total volume of the sample extraction solution (mL), N is the dilution ratio of the sample extraction solution, and m is the sample mass (g).

### 2.4. Measurement of Total Starch and Amylose Content

The total starch and amylose contents were determined according to the manufacturer’s instructions for the reagent kit (Beijing Solaibao Biotechnology Co., Ltd., Beijing, China).

### 2.5. Protein Content and Vitamin C (VC) Content

Soluble protein and VC contents were determined according to the manufacturer’s instructions for the reagent kit (Nanjing Jiancheng Biotechnology Research Institute, Nanjing, China).

### 2.6. Measurement of Crude Fat Content

Crude fat in lotus seeds refers to GB/T 14772-2008 [18] “Determination of crude fat in food”. Each sample was analyzed in triplicate.

### 2.7. Measurement of DPPH Radical Scavenging Capacity

DPPH radical scavenging ability was measured according to a previously described method, with slight modifications [19]. Lotus seed flesh (0.5 g) and lotus plumule (0.5 g) were accurately weighed, 5 mL of anhydrous ethanol separately was added, shaken for 2 min, sonicated at 50 °C for 30 min, centrifuged at 10,000 r/min at 4 °C for 10 min, and the supernatant was collected for later use. The supernatant was diluted nine times with anhydrous ethanol. Two milliliters of diluent and 0.2 mmol/L MixDPPH solution were mixed in a shaded reaction for 30 min. Absorbance was measured at 517 nm. The experiment was repeated three times using anhydrous ethanol as a blank control, and the DPPH free radical scavenging rate of lotus seeds was calculated according to the following formula:

(2) $$\mathrm{DPPH}\;\mathrm{radical}\;\mathrm{scavenging}\;\mathrm{ability}(\%)=[1-\frac{A_{0}-A_{1}}{A_{2}}]\times 100\%$$

where A_0_ represents the absorbance of the sample and the DPPH solutions (2 mL each). A_1_ is the absorbance of the sample and the anhydrous ethanol solutions (2 mL each). A_2_ is the absorbance of DPPH and the anhydrous ethanol solutions (2 mL each).

### 2.8. Measurement of Alkaloids in Lotus Plumule

The alkaloid content was determined using reference methods with slight modifications [20,21]. Then, 2 g of lotus plumule powder was diluted to 25 mL with methanol solution, sonicated for 30 min, heated and condensed for reflux for 30 min, weighed after cooling, made up for the loss of mass with methanol solution, filtered through a 0.45 μm filter membrane, and the filtrate was injected. The contents of liensinine, isoliensinine, neferine, and lotusine were determined using a high-performance liquid chromatography (HPLC) system equipped with a photo-diode array (PDA) detector, with a detection wavelength of 282 nm and an Eclipse XDB-C18 column (4.6 × 250 mm, 5 µm), column temperature of 30 °C, mobile phase of acetonitrile/50 mmol/L triethylamine aqueous solution (*v*/*v*), and an injection volume of 20 µL. The four alkaloids in the lotus plumule were quantified by measuring the peak time and peak area of a mixed standard solution of the four alkaloids.

### 2.9. Statistical Analysis

All tests were performed in triplicate. The results and significance (*p* < 0.05) are displayed using analysis of variance, and the results are expressed as mean ± standard deviation (SD). All data were analyzed using Statistical Package for the Social Sciences software (version 26; SPSS Inc., Chicago, IL, USA) and Microsoft Excel software (version 2016; Redmond, WA, USA). All graphs were plotted using Origin software (version 2019; Origin Lab, Northampton, MA, USA).

## 3. Results

### 3.1. Analysis of Protein and Soluble Sugar Content

**Figure 1:**
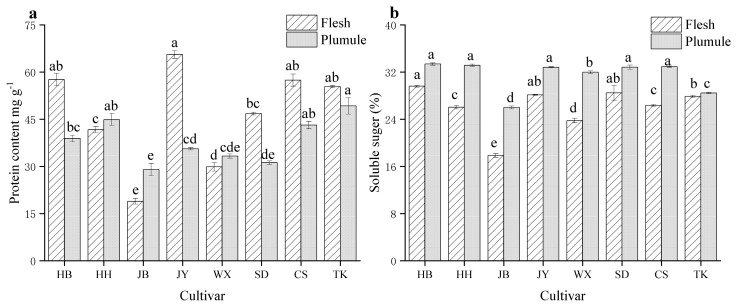
Protein content (**a**) and soluble sugar content (**b**) of different cultivars and parts of lotus seeds. Different lowercase letters indicate significant differences (*p* < 0.05).

Soluble sugars and proteins are important reference factors for physiological indicators and nutritional quality of fruits and vegetables [22,23]. As depicted in Figure 1a, there were significant differences in protein content between the flesh and plumule of the different varieties of lotus seeds (*p* < 0.05). In the flesh, except for HH, JB, and WX, the soluble protein content of the other varieties was higher than that in the lotus plumule. The protein content in JY flesh (34.37 mg/g) was higher than that in the other varieties. In Lian’s plumule, TK, CS, and HH had higher protein content and, overall, had the highest JY content (53.04 mg/g); JB had the lowest overall protein content (25.12 mg/g). After harvesting, lotus seeds undergo protein denaturation and hydrolysis, and the proteins in the organelles are continuously released into free proteins [24]. Differences in the protein content among different varieties of lotus seeds can lead to different rates of free protein production and varying levels of soluble protein.

The soluble sugar content is linked to the taste of lotus seeds. Some of these monosaccharides are important flavor substances that affect the freshness and sweetness of lotus seeds during growth and development. When its content is low, lotus seeds have an astringent taste [10]. There was a significant difference in the soluble sugar content between the flesh and plumule of the different varieties of lotus seeds (*p* < 0.05). The soluble sugar content in all eight varieties of lotus plumules, except TK and JB, was higher than 30%. HB (33.37%) had the highest soluble sugar content in lotus plumules. The soluble sugar content in the JB flesh and lotus plumule was the lowest at 17.87% and 26.01%, respectively (Figure 1b). The differences in the soluble sugar content among different cultivars of lotus seeds may be related to the water stress they experience during growth [25].

### 3.2. Analysis of Crude Fat Content

**Figure 2:**
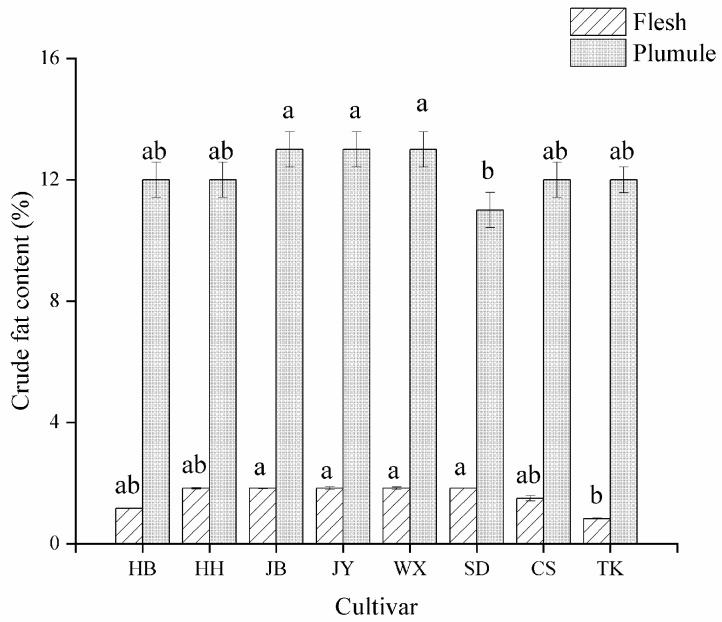
Crude fat content in different cultivars and parts of lotus seeds. Different lowercase letters indicate significant differences (*p* < 0.05).

The crude fat content can reflect the nutritional value of fruits and vegetables. A higher fat content indicates greater energy provision, and varieties with higher crude fat content may appear glossier [26]. The differences in crude fat content among the various fruit varieties were insignificant, with a flesh content of approximately 2% (Figure 2), which is consistent with the findings of Luo et al. [14]. The crude fat content of the lotus plumule was approximately 12% (Figure 2), which is significantly higher than flesh (*p* < 0.05), which is because the lotus plumule, as the embryo of lotus seeds, stores energy for growth and development and is rich in oil [27].

### 3.3. Analysis of VC Content

**Figure 3:**
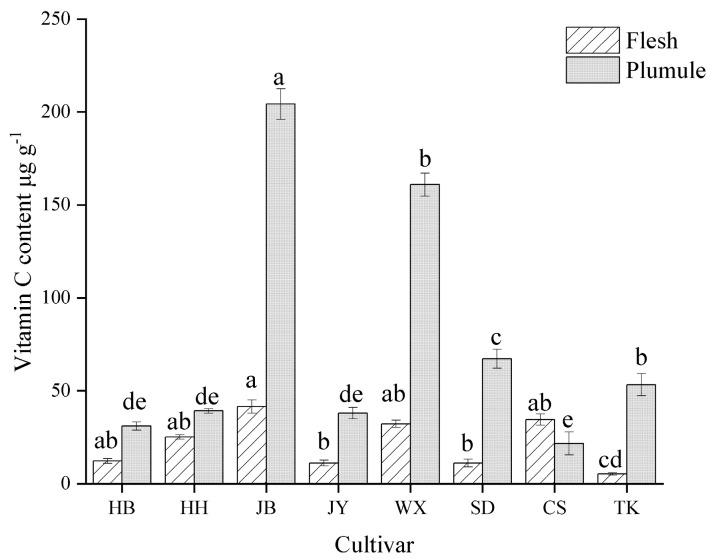
VC content in different cultivars and parts of lotus seeds. Different lowercase letters indicate significant differences (*p* < 0.05).

VC is an important antioxidant that inhibits superoxide production and reduces stress responses [28]. As depicted in Figure 3, all lotus seed varieties, except CS, had significantly higher VC content in the plumule than in the flesh (*p* < 0.05) among the varieties. The JB (219.51 mg/g) and WX (170.34 mg/g) had significantly higher VC contents in the lotus plumules than the other varieties. The CS lotus plumule had the lowest VC content (12.29 mg/g); however, its flesh contained higher VC levels than those of most other varieties. TK had the lowest VC content in flesh (5.27 mg/g). Overall, JB and WX varieties exhibited higher VC contents. Gonz á lez Centeno et al. demonstrated that agricultural and sideline products with antioxidant activity possess significant application potential and are more conducive to development value [29]. Therefore, JB and WX are advantageous for the development of products, such as lotus seed juice and whitening oral liquids.

### 3.4. Differences in Total Starch and Amylose Content

**Figure 4:**
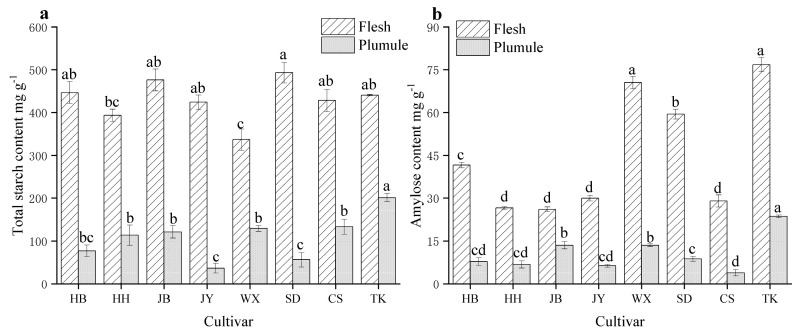
Total starch content (**a**) and amylose content (**b**) of different cultivars and parts of lotus seeds. Different lowercase letters indicate significant differences (*p* < 0.05).

Starch is a crucial indicator of lotus seed quality and is the largest nutritional component of lotus seeds. Lotus seed starch, a high amylose-specific starch, has an aging rate closely related to the quality of lotus seeds. It is also an important functional ingredient for various functional foods and processing applications [15]. Starch primarily exists in the flesh. As displayed in Figure 4a, SD had the highest total starch content (493.01 mg/g). The differences in total starch content among HB, JY, CS, and TK were insignificant, whereas WX had the lowest total starch content (336.93 mg/g). In the plumule, the TK variety had the highest total starch content (200.98 mg/g), whereas the JY variety had the lowest (36.27 mg/g).

The amylose content is related to the quality of grains and is an important indicator of cooking quality. It is directly related to the viscosity, water absorption, and swelling properties of grains [30]. The eight varieties of amylose demonstrated significant differences (*p* < 0.05). The amylose content in the flesh of WX, TK, and SD fruits was relatively high (Figure 4b). Additionally, TK and WX lotus plumules exhibited higher amylose contents than the other varieties (Figure 4b). Consequently, both varieties have a hard and aged taste. Total starch is primarily categorized into two types: linear and branched. Despite having a low total starch content, the WX variety has a high amylose content. This may be due to the high content of starch debranching enzymes or the oxidation and hydrolysis of most of the amylose in lotus seeds into amylose, making it less prone to gelatinization [31,32].

### 3.5. Total Phenolic Content, Flavonoid Content, and DPPH Antioxidant Capacity of Different Varieties of Lotus Seeds

**Figure 5:**
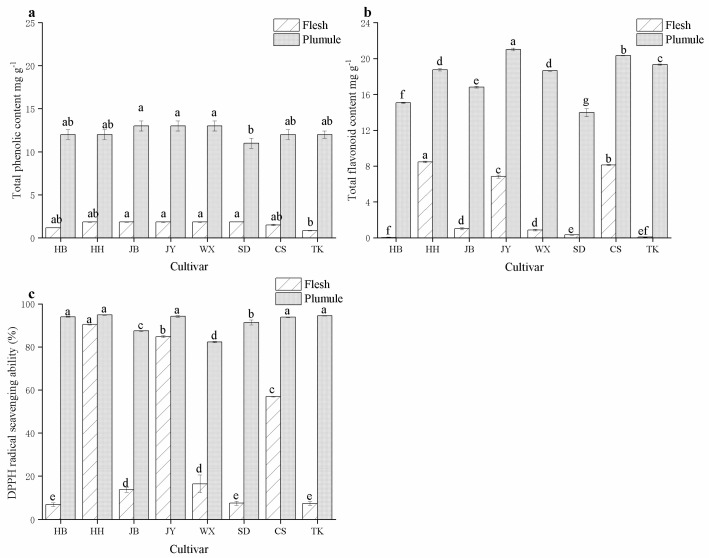
Total phenolic content (**a**) flavonoid content (**b**) and DPPH radical scavenging ability (**c**) of different cultivars and parts of lotus seeds. Different lowercase letters indicate significant differences (*p* < 0.05).

As illustrated in Figure 5, there were significant differences (*p* < 0.05) in the clearance rates of phenolic and flavonoid substances and DPPH radical scavenging abilities among the different varieties and parts of lotus seeds. Phenolic and flavonoid substances were primarily present in the lotus plumule. Total phenols contribute to free radical clearance and anti-aging and antioxidant properties [33]. Figure 5a displays that the total phenolic content in the eight varieties of lotus seeds and lotus plumules exceeded 12 mg/g. JB, JY, TK, and other varieties had higher total phenolic content, while the SD lotus plumule had the lowest (12.88 mg/g). In contrast, the phenolic content in the flesh of HB, SD, and TK was relatively low, which was significantly different from that in the lotus plumule. Total phenolic content is crucial for the DPPH radical scavenging ability of lotus seeds. As presented in Figure 5c, the DPPH radical scavenging ability of the lotus plumule exceeded 80%. The DPPH radical scavenging abilities of HH, JY, and CS in the flesh were relatively high, mirroring the total phenolic content in the flesh. This indicates that a higher total phenolic content correlates with a stronger DPPH radical scavenging ability [34].

Flavonoids possess biological activity and medicinal value, including anti-inflammatory, anticancer, and antioxidant effects [35,36]. The flavonoid content of the lotus plumule was significantly higher than that of the flesh. Among them, the flavonoid contents of HH, JY, and CS are higher than those of other varieties (Figure 5b) However, the flavonoid contents in the flesh of HB (0.01 mg/g) and TK (0.09 mg/g) are extremely low. Additionally, there were similar variations in the flavonoid content, total phenolic content, and DPPH radical scavenging ability among the different varieties.

### 3.6. Differences in Alkaloid Content

**Figure 6:**
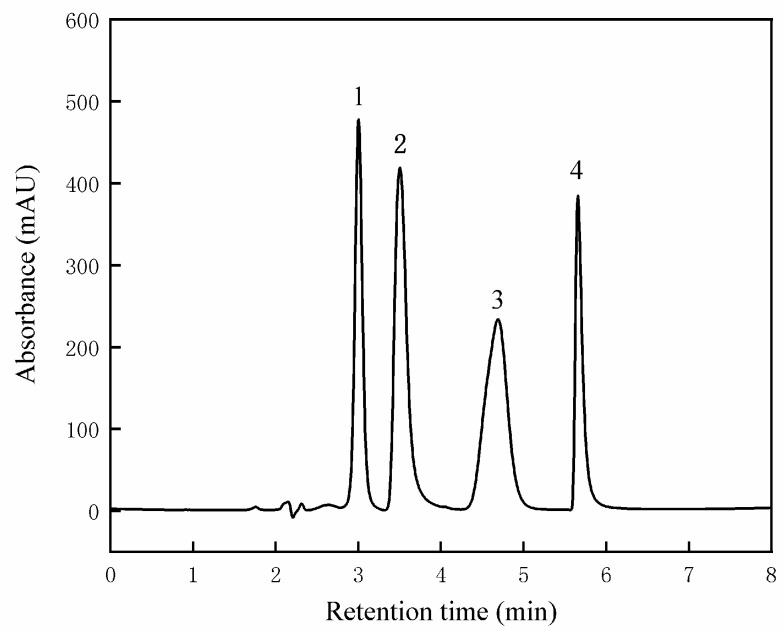
HPLC chromatogram of lotus plumule alkaloid mixed standard. 1 represents liensinine, 2 represents isoliensinine, 3 represents neferine, and 4 represents lotusine.

**Figure A1:**
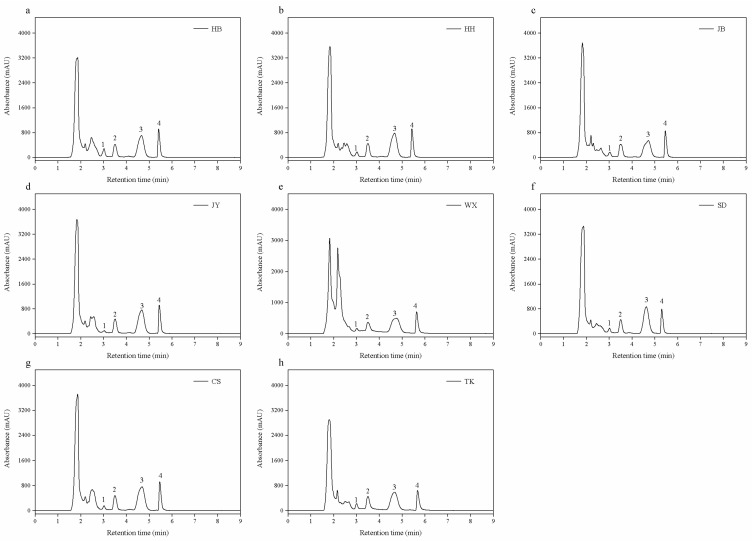
HPLC chromatogram of eight lotus seed cultivars. 1 represents liensinine, 2 represents isoliensinine, 3 represents neferine, and 4 represents lotusine. HH chromatogram (**a**), HH chromatogram (**b**), JB chromatogram (**c**), JY chromatogram (**d**), WX chromatogram (**e**), SD chromatogram (**f**), CS chromatogram (**g**), and TK chromatogram (**h**).

Lotus plumule quaternary ammonium alkaloids can also be used to treat neurological diseases [37]. These unique functional active substances in the lotus plumule are the main sources of bitter components in the lotus seeds. The primary alkaloid components of lotus seeds are liensinine, isoliensinine, neferine, and lotusine [38]. The HPLC chromatogram of the mixed alkaloid standard is presented in Figure 6, and the HPLC chromatograms of eight lotus seed cultivars are shown in Figure A1 (Appendix A). There were significant differences (*p* < 0.05) in the alkaloid content among the eight varieties of lotus seeds. The main alkaloid content of the different varieties is presented in Table 1. HB had the highest content of liensinine (0.93 mg/g), followed by TK (0.6 mg/g) and HH (0.49 mg/g), while JY contained only 0.15 mg/g. TK had the highest content of isoliensinine (3.22 mg/g), whereas WX had the lowest (2.65 mg/g), although there was no significant difference compared to SD and HB (*p* > 0.05). The content of neferine in CS was higher than other varieties (9.47 mg/g), while the content of JB was the lowest (6.96 mg/g). Compared with other varieties, HH, JY, and SD exhibited certain advantages in neferine content. The CS content is the highest (7.09 mg/g) in lotusine. Except for WX (5.88 mg/g) and SD (5.91 mg/g), the ammonium alkaloid content in other lotusine varieties was higher than 6 mg/g. Overall, the neferine content was the highest, followed by isoliensinine and lotusine. The liensinine content was the lowest, and CS, HH, and JY had relatively high contents among the four alkaloids.

## 4. Discussion

Many studies on the differences between fruit and vegetable varieties have focused on chemical components, such as protein, starch, sugar, and vitamins. Lotus seed protein has good nutritional value but can cause conformational and structural changes during processing [39], affecting the quality of processed foods. Varieties with a higher protein content, such as CS, JY, and TK, can maintain a high protein level even after processing, making them more suitable as functional products. Soluble sugars, including monosaccharides, such as glucose and fructose, are important indicators of the sweetness of lotus seeds. The soluble sugar content in the lotus plumule and flesh of HB, SD, and JY was higher, resulting in sweeter flavors. VC is an essential nutrient for the human body and is mainly present in its reduced form in fresh fruit and vegetables. The VC content of the lotus plumule was higher than that of the flesh. Specifically, JB and WX had significantly higher VC contents than the other lotus seed varieties. While the VC content in lotus seed flesh and plumule is higher than that determined by Arooj et al. [6], which can meet daily VC needs. Lipids, the main source of unsaturated fat, are closely related to improved cholesterol levels [40]. The crude fat content of flesh (1–2.5%) is similar to the research results of Bhat et al. (3.68%) [41]. The lotus plumule, developed from the embryonic root, has a significantly higher fat content than the flesh. Varieties such as TK and SD have lower crude fat content than other varieties, making them more suitable for individuals with abnormal blood lipid levels.

Starch content is linked to the aging of lotus seeds [42]. A higher starch content can increase the hardness of lotus seeds, indicating a strong metabolic activity after harvesting. Amylose content is an important indicator of lotus seed processing, as it is positively correlated with moisture absorption and expansion rates of processed lotus seeds and negatively correlated with viscosity and color [14]. JB and CS had higher total starch and lower amylose content. This may be due to the presence of starch debranching enzymes or the varying ability of branched starch to hydrolyze into amylose [31,32]. Consequently, JB and CS are more suitable for cooking and consumption because of their low amylose content, making them ideal raw materials for food conditioning. In contrast, high-amylose varieties such as TK and WX are more suitable for producing baked goods and noodles containing resistant starch [15].

Lotus seeds contain abundant polyphenolic compounds, including polyphenols such as catechins, gallic acid, chlorogenic acid, quercetin, and isoquercetin [4]. Research findings on various lotus seed varieties and parts show that the lotus plumule contains significantly higher total phenolic and flavonoid contents than the flesh. The total phenolic content in the plumule was comparable to that reported for lotus seeds in the literature, but the content in the flesh is slightly lower [9]. Similarly, the flavonoid content across different lotus seed varieties aligns with previous reports (930–1595.86 mg/100 g) [3]. High levels of phenolic compounds exhibit potent functional activities that positively influence the biological activity and therapeutic potential. TK, JY, CS, and HH exhibited elevated levels of these phenolic compounds, making them suitable raw materials for medicinal or health food processing. Moreover, except for WX, the DPPH radical scavenging ability of lotus plumules exceeds 90%, significantly higher than that of flesh, indicating that lotus plumules have stronger antioxidant activity, which is beneficial to health. The DPPH radical scavenging ability is similar to the results of Kim and Shin [43]. The antioxidant properties of different lotus seeds may be related to their biochemical components, particularly secondary metabolites such as phenolic compounds. DPPH radical scavenging ability correlates closely with the content of phenolic substances [44,45], as indicated by the results of this study.

The main alkaloids discovered in the plumule of lotus include liensinine, isoliensinine, neferine, and lotusine [46], which have antioxidant, blood pressure-lowering, antibacterial, and anti-inflammatory effects. However, these alkaloids also impart a bitter taste to lotus seeds, which can diminish their overall palatability [38]. CS, HH, and JY had the highest contents of main alkaloids and phenolic compounds, indicating that these three varieties have stronger functional activity, but their taste may be more bitter. Tu et al. demonstrated that even during the optimal harvesting period, fresh lotus plumules retained slight bitterness [9]. Techniques for lotus seed processing can improve bitterness. Xu et al. showed that chitosan effectively inhibits the bitterness caused by alkaloids and flavonoids at the molecular level [47]. Li et al. discovered that amphiphilic block copolymers mask the bitterness of alkaloids: berberine hydrochloride, matrine, and gentiopicroside [48]. Consequently, during lotus plumule processing, coating, chemical modification, and microencapsulation can be employed to reduce the bitterness of the lotus plumule, making it easier for consumers to accept it.

## 5. Conclusions

This study found that there were differences in the nutritional and functional active substances of different varieties and parts of the lotus seeds. The results demonstrated that higher levels of soluble protein and soluble sugar, as well as lower levels of starch, result in a better and fresher quality of lotus seeds. Higher levels of phenolic substances and alkaloids would give lotus seeds stronger functional activity. JY flesh contains high contents of soluble proteins, soluble sugars, and crude fat. The JB and CS lotus plumule had higher VC content. The total starch and amylose contents of SD and TK were relatively high and prone to aging, whereas HH and JB had the lowest amylose content, which is more conducive to the deep processing of lotus seeds. Among the main nutritional components, except for soluble protein, total starch, and amylose, the content of lotus plumule is higher than that of flesh. The content of functional active substances in lotus plumule is also significantly higher than that in flesh. JY and CS have higher levels of phenolic substances, flavonoids, and alkaloids; therefore, they have stronger antioxidant properties compared to other varieties. This study lays the foundation for the deep processing of lotus seeds and the rapid differentiation technology of varieties. The direction of processing advancement is to alleviate and prevent the occurrence of chronic diseases by separating and extracting bioactive substances that are beneficial to health. The differences in nutrition and functional active substances of lotus seeds from different varieties and parts also provide better dietary guidelines for people. This study may open up a new research field for other researchers to explore potential plant chemicals with nutritional importance from the flesh and plumule of lotus seeds. In the future, different edible or medicinal products can be developed based on the differences in nutrients and functional active substances of different varieties of lotus seeds, further exploring the potential of lotus seeds as antioxidants.

## Figures and Tables

**Table 1:** Alkaloid content in different lotus seed varieties (mg/g). Different lowercase letters indicate significant differences (*p* < 0.05).

Cultivar	HB	HH	JB	JY	WX	SD	CS	TK
Liensinine	0.93 ± 0.02 ^a^	0.49 ± 0.01 ^c^	0.48 ± 0.01 ^c^	0.15 ± 0.01 ^g^	0.30 ± 0.01 ^f^	0.42 ± 0.01 ^d^	0.37 ± 0.00 ^e^	0.6 ± 0.02 ^b^
Isoliensinine	2.69 ± 0.03 ^d^	2.89 ± 0.01 ^c^	3.06 ± 0.00 ^b^	2.96 ± 0.01 ^c^	2.65 ± 0.01 ^d^	2.67 ± 0.01 ^d^	3.08 ± 0.01 ^b^	3.22 ± 0.08 ^a^
Neferine	7.87 ± 0.00 ^e^	8.89 ± 0.01 ^b^	6.96 ± 0.00 ^h^	8.63 ± 0.01 ^d^	7.02 ± 0.04 ^g^	8.73 ± 0.00 ^c^	9.47 ± 0.01 ^a^	7.42 ± 0.04 ^f^
Lotusine	6.95 ± 0.01 ^c^	7.02 ± 0.01 ^b^	6.35 ± 0.01 ^d^	6.94 ± 0.01 ^c^	5.88 ± 0.03 ^f^	5.91 ± 0.00 ^f^	7.09 ± 0.01 ^a^	6.08 ± 0.03 ^e^

## Data Availability

The original contributions presented in the study are included in the article, further inquiries can be directed to the corresponding author.

## Appendix A

The HPLC chromatograms of eight lotus seed cultivars are shown in Figure A1.
